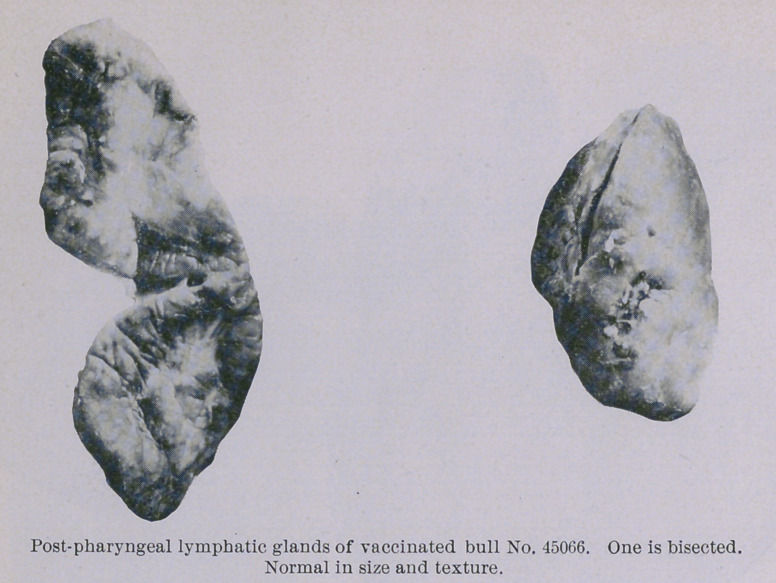# Some Experiments upon the Immunization of Cattle against Tuberculosis1Read before the Pathological Society of Philadelphia, November 13, 1902.

**Published:** 1902-11

**Authors:** Leonard Pearson, S. H. Gilliland

**Affiliations:** State Veterinarian of Pennsylvania; Assistant Bacteriologist of the State Live-Stock Sanitary Board


### THE JOURNAL

OF

### COMPARATIVE MEDICINE AND VETERINARY ARCHIVES.

VOL. XXIII. *NOVEMBER, 1902.* No. 11.

### SOME EXPERIMENTS UPON THE IMMUNIZATION OF CATTLE AGAINST TUBERCULOSIS.[1]

BY LEONARD PEARSON, B.S., V.M.D.,
STATE VETERINARIAN OF PENNSYLVANIA,

AND

S. H. GILLILAND, V.M.D ,
ASSISTANT BACTERIOLOGIST OF THE STATE LIVE-STOCK SANITARY BOARD.

(From the Laboratory of the State Live-stock Sanitary Board of Pennsylvania).

WHEN an extensively tubercular herd is tested with tuberculin one usually finds some animals that do not react to the test and are free from disease. These uninfected animals may be young or they may be recent additions to the herd, and their freedom from disease may be due merely to the fact that they have not had time to contract it ; on the other hand, they are often cows that have been members of the herd and exposed to infection for years. That the freedom of these cattle that have long resisted the disease is not due to breed or family immunity has, in numerous instances, been shown by the fact that their parents or offspring have succumbed to tuberculosis.

To what is such resistance to tuberculosis due? It is evident that it does not depend upon species, breed, or lack of exposure. It is an individual factor. An animal may possess some power within itself to resist the tubercle bacilli that it is constantly exposed to and must daily inhale and ingest.

Careful observation of these cattle and study of them in series show that the immunity they possess is not due to what is roughly termed good general health or what the stockman knows as good

[1] Read before the Pathological Society of Philadelphia, November 13, 1902.

condition. Cattle resistant to tuberculosis may suffer with some other disease or be in a bad state of nutrition. Cattle that contract tuberculosis show, in very many instances, until the infection is well advanced, the usual signs of good health, such as soft coat, pliable skin, clear eyes, good appetite, and regular growth or increase of weight or yield of milk in proportion to the quantity and quality of food consumed. It appears, then, that there is reason to believe that some cattle have a specific resistance to tuberculosis. We know that specific resistance or immunity of the individual, occurring under natural conditions, usually depends on a previous attack of the disease against which the animal is immune, or, as in the case of Texas fever, upon the continued existence of the disease in a form so mild as not to appreciably disturb the various functions. This principle receives practical application when persons are rendered immune to smallpox or animals to anthrax, black-quarter, lung plague, rabies, or Texas fever by inoculating them with the attenuated but living virus of the respective disease, and thus causing them to develop it in a comparatively mild form, from which speedy recovery and subsequent immunity are almost certain.

From the inoculation there results the automatic development of an antitoxin that counteracts the toxin of the disease, and, at the same time, prevents or retards the growth of the organism of that disease. Until comparatively recently this principle has been thought to hold only in respect to certain acute infectious diseases, but it is now known to be of much wider application. Protection upon this principle is usually known as vaccination. In some cases the germ-free toxin is used for a similar purpose.

In 1901 we conducted an experiment for the purpose of determining the influence of Koch's original tuberculin upon the resistance of cattle to tuberculosis. In this experiment were used four cows known by the numbers 26554, 26555, 26556, and 26557. Each was tested with tuberculin before it was admitted into the experiment. Two of these cows, 26554 and 26557, were given daily injections of 5 c.c. of concentrated tuberculin for ten days, from August 24 to September 2, 1901, inclusive. Each of the four cows in the experiment was fed daily 100 grammes of hacked tuberculous lung tissue from a cow, for ten days, from the 10th to the 19th of September, inclusive. The first pair of cows, 26554 and 26557, that had received preliminary injections of tuberculin were given subcutaneously 15 c.c. of concentrated tuberculin each day during the progress of the feeding upon tuberculous material.

The other two cows, 26555 and 26556, which had not received the daily preliminary injections of tuberculin, received no tuberculin during the experiment.

One of the cows (26554) that had been treated with tuberculin, and one (26555) that had not been treated with tuberculin were killed November 25, 1901. The cow (26554) that had been treated with tuberculin showed upon post-mortem examination lesions of tuberculosis in the post-pharyngeal and mesenteric lymphatic glands. The control cow (26555) showed lesions of tuberculosis in the right lung and in the bronchial and mediastinal lymphatic glands, the post-pharyngeal and intermaxillary lymphatic glands and in the mesenteric lymphatic glands. The lesions in this control cow were more widely distributed and more advanced than in the cow that had received large quantities of tuberculin.

The other two cows of the experiment were killed December 16, 1901. In the first of these (26557) which had received the injections of tuberculin, no lesions of tuberculosis were found excepting in the mesenteric lymphatic glands. A few of these glands of both the small and large intestine showed small areas of caseation. The second control cow (26556) showed lesions of tuberculosis in both lungs, the bronchial, mediastinal and post-pharyngeal glands; and the lymphatic glands of the mesentery were more extensively involved than in the preceding cow.

From this it would appear that subcutaneous injections of the toxin of the tubercle bacillus had had some influence in increasing the resistance of these two cows to feeding tuberculosis.

E. A. de Schweinitz reported in the *Medical News* for December 8, 1894, some experiments made by him upon guinea-pigs, in which these animals were inoculated with tubercle bacilli of human origin cultivated for about twenty generations upon glycerin beef broth, and were afterward inoculated with tuberculous material from a cow. The guinea-pigs so treated remained free from tuberculosis, while check animals inoculated with the same tuberculous material from the cow died of tuberculosis within seven weeks. De Schweinitz also showed that the twentieth generation of broth culture appeared to be incapable of producing tuberculosis in a cow when she was inoculated intravenously with a small quantity. De Schweinitz and Schroeder report (U. S. Dept. of Agr., *B. A. I. Bulletin*, No. 13, 1896) upon other inoculations similar in nature and confirmatory of the above results. They show, further, that the attenuated culture they were working with was not virulent for cattle when inoculated intravenously in quantities of 500 c.c. of suspension in liquid.

The immunizing effect upon cattle of the administration intravenously of tuberculous material or of living cultures has been studied by J. McFadyean and by von Behring.

McFadyean reported in the *Journal of Comparative Pathology and Therapeutics* for June, 1901, and March, 1902, upon some experiments regarding the immunization of cattle against tuberculosis. He inoculated four cattle intravenously with emulsions of tuberculous material and cultures from various sources. One of these cattle, which had responded to the tuberculin test, and was, no doubt, tubercular upon the beginning of the experiment, was given about 150 c.c. of tuberculin in divided doses before inoculation. Fifteen weeks after inoculation this animal was killed and was found to contain but one tubercle, the size of a pea and completely calcified, in a mesenteric gland. Two control cattle inoculated with an equal dose of the same material died of generalized tuberculosis. Of the other three cattle of the series one was tubercular at the beginning of the experiment. All of these were inoculated intravenously from seven to eleven times during a period of from two to three years with emulsions of tuberculous materials and with cultures from various sources. It is interesting to note that the first inoculation upon each of the cows that was free from tuberculosis at the beginning of the experiment was made with avian material which was probably of very low virulence for cattle. The cattle so inoculated died of tuberculosis after two to three years from the beginning of the experiment, and in each case the chief lesions were in the kidneys and the brain or its covering membranes. The cerebral lesion appears to have been the immediate cause of death in each instance. There can be no doubt that these animals were remarkably resistant to tuberculosis, because they lived for months or years after repeated inoculations with large quantities of material of proven virulence for cattle.

Von Behring announced December 12, 1901, that he was engaged in studying the immunization of cattle against tuberculosis, and he has since published a report (*Beiträge zur Experimentellen Therapie*, Heft 5, 1902) upon his work. He details experiments upon several cattle treated with injections of tuberculin and with cultures of varying degrees of virulence and from several sources, and also inoculated with tuberculous material or cultures of proven virulence. It may be noted that a pure culture virulent for cattle was not available for use in von Behring's work until 1901. The experiments upon seven cows specially reported were commenced between July and December, 1901. These cows have all received repeated injections of tuberculin and of tubercle virus of low and high virulence. All of the protected cows are still alive excepting one that was killed and was found to have numerous tubercular nodules in the lungs, although these were believed to be retrogressive. This general experiment cannot be looked upon as finished, and any report upon it must be regarded as incomplete until the cows die or are killed and are examined post-mortem. The cows may appear to be in good health now, but, notwithstanding, they may be extensively tubercular. However, that they are alive after receiving quantities of virulent tuberculous material that are sufficient to kill untreated cows shows that they have extraordinary resistance to tuberculosis. The method used to treat these cows was not systematic nor the one that he now recommends upon the evidence of experiments not yet published. The method now recommended by him is to inject intravenously 0.001 gramme of a scraping from a serum culture of tubercle bacilli dried in vacuum, powdered, and suspended in water. The culture used for this purpose was obtained originally from human sputum and has been grown in his laboratory since 1895. After four weeks a second injection is made containing twenty-five times the original quantity of tubercle bacilli, or 0.025 gramme. Von Behring has now underway extensive experiments planned to test the resistance of immunized calves to natural infection from association with infected animals in contaminated premises.

Since 1896 tuberculosis of cattle has been the subject of special and extensive study and experimentation in the laboratory and research station of the Pennsylvania State Live-stock Sanitary Board. During this time the virulence for cattle and other animals of tubercle culture and material from many sources have been tested by Dr. M. P. Ravenel, Dr. John J. Repp, and ourselves. The results of some of this work have been reported upon several occasions to this Society by Dr. Ravenel and to the British Congress on Tuberculosis in 1901. Some experiments looking toward the development of better methods for repressing tuberculosis in herds have been reported by Dr. Leonard Pearson.

It has been shown by numerous experiments that the sputum of persons suffering from consumption and cultures of tubercle bacilli made from such sputum are usually comparatively non-virulent for cattle. It is important to know, further, that a given culture of sputum tubercle bacilli is incapable of producing serious disease in such quantities as it may be necessary to use in an attempt to increase an animal's resistance to tuberculosis.

The following experiment throws light upon the question as to the quantity of culture of this kind that may be administered and the effect of repeated inoculations made in four different ways. A Jersey heifer (26415) shown by tuberculin test to be free from tuberculosis was inoculated intraperitoneally September 29, 1900, with 4 c.c. of a standard suspension[1] of human sputum culture that had remained in a collodion capsule in the abdominal cavity of a bull for seven months, and was then regained in pure culture by Dr. Ravenel. The third generation on blood serum furnished the material for this inoculation. The heifer was inoculated intravenously March 15, 1901, with 13.5 c.c. of a standard suspension of tubercle bacilli, probably of human origin, that had passed through a coati (*Nasua narica*), and were recovered in pure culture by Dr. Theobald Smith in 1895. This culture had afterward remained about one year in a collodion capsule in the peritoneal cavity of a heifer, had been recovered by Dr. Ravenel, and the third generation on blood serum after recovery supplied the material for the present inoculation. A second intravenous inoculation with 10 c.c. of similar suspension was made June 1, 1901. August 23, 1901, this heifer was inoculated with 20 c.c. of a standard suspension in water of a culture (H) of tubercle bacilli from human sputum. This quantity of material was divided into four parts of 5 c.c. each, and these parts were injected beneath the skin, into the peritoneal cavity, into the jugular vein, and into the lung, respectively. These injections were repeated at intervals of from seven to ten days until January 29, 1902. The quantity of standard suspension was increased 10 c.c. with each successive inoculation, so that at the last, the eighteenth, inoculation the total quantity given was 160 c.c. The total quantity given in this series of inoculations was 1797 c.c. of standard suspension. There was a rise of temperature of from two to four degrees following each inoculation after the first one. The first inoculation caused no temperature reaction. The heifer was in strong, thrifty condition at the completion of the series of inoculations, and improved in condition throughout the following months. It was

[1] By a standard suspension is here meant a suspension of tubercle bacilli in water in such quantity as to give an opacity equal to that of a twenty-four-hour culture of typhoid bacilli in bouillon. 1 c.c. of such a suspension is estimated to contain the equivalent of 0.0013 gramme of tubercle bacilli after drying ten days in a desiccating chamber over calcium chloride.

killed August 14, 1902. The condition was good, and there was an abundance of fat upon the carcass and about the intestines. The post-mortem examination revealed the lungs to be normal in color and elastic; they were free from nodules, but were attached to the chest walls along the lower borders by fibrous bands. A few flakes of fibrin were found upon the omentum, and these flakes contained a few calcareous nodules about one-twelfth of an inch in diameter. The liver was adherent to the diaphragm over an area five inches in diameter.

A yearling grade short-horn bull (26442) after having been tested with tuberculin and proven to be free from tuberculosis, was inoculated intraperitoneally November 19, 1900, with 16 c.c. of a suspension of tubercle bacilli from a culture from human sputum that had remained in a collodion capsule in the peritoneal cavity of a bull for seven months. The third generation on blood serum after recovery furnished the material for this inoculation. March 17, 1901, this bull was inoculated intravenously with 13.5 c.c. of a standard suspension of a culture similar to that used in the inoculation of the above heifer (26415) on March 15 and June 1, 1901. This animal was subsequently inoculated in the same manner as the heifer, receiving eighteen inoculations between August 23, 1901, and January 10, 1902. He received in all 1710 c.c. of standard suspension. He reacted following the inoculations very much as the heifer, although somewhat more slowly. He remained in good condition and apparent good health until he was killed, excepting for the development of an abscess over the jugular vein, which was opened November 22d, and afterward healed nicely. January 18, 1902, this bull was inoculated intraperitoneally with 10 c.c. of a standard suspension of tubercle bacilli from a culture (H) of bovine origin. The virulence of this culture for cattle had been proven by numerous inoculations, among which the following may be mentioned: A cow (26431) weighing 950 pounds was inoculated intravenously January 8, 1901, with 5 c.c. of a standard suspension from a culture of bovine tubercle bacilli H. The cow lost weight rapidly to 750 pounds, and died March 4, 1901. Post-mortem examination revealed most extensive miliary tuberculosis of the lungs. Another cow (26433), weighing 698 pounds, was similarly inoculated at the same time, and died January 26th of miliary tuberculosis of the lungs. This cow received two injections of tuberculin of 0.4 c.c. each on January 16th and 22d. Both of these cows had been shown to be free from tuberculosis by tuberculin test before they were inoculated. A red heifer (45072), about eight months old, was tested and did not react. It was inoculated intraperitoneally April 30, 1902, with 5 c.c. of standard suspension of bovine culture H. It died June 7, 1902, and was found to contain extensive lesions of tuberculosis upon the peritoneum and abdominal organs, and the lungs, also, were crowded with small tubercles. The bull (26442) was killed August 13, 1902. The general condition was good, and there was much fat upon the carcass and about the internal organs. The pleura lining the lower half of the chest was covered by a sheet of partly organized fibrin from one-eighth to one-third of an inch thick. The lungs themselves contained a few nodules about one-half inch in diameter surrounded by thick walls and containing caseous pus in which there were many tubercle bacilli. These nodules did not seem to be progressive, and appeared to be abscesses indicating the sites of previous inoculations. The peritoneum covering the abdominal walls, the stomach, intestines, spleen, and liver was coated with a layer of partly organized fibrin, as in the chest. The lymphatic glands about the rectum were enlarged and caseous. The surface of the omentum was rough from the presence of a thin layer of partly organized fibrin. The omentum was thickened in places, but there were no distinct nodules. From the fact that the fibrinous coating of the serous membranes was as pronounced in the thoracic as in the abdominal cavity it is probable that the virulent culture of tubercle bacilli injected into the abdomen has little to do with the production of this deposit, which may readily have resulted from the discharge of a pulmonary abscess into the pleural cavity or the discharge into the peritoneal cavity of the purulent contents of one of the softened lymphatic glands in the pelvis.

It is evident that the sputum tubercle bacilli used for the inoculation of these two animals (26415 and 26442), even in the exceedingly large quantities in which they were employed, were incapable of causing general tubercular infection. Even the intraperitoneal inoculation of the bull with a quantity of virulent culture nearly twice as great as was necessary when similarly administered to kill an unprotected heifer did not, so long as he was permitted to live, appreciably disturb his general health. The human sputum culture M used for these inoculations was obtained by Dr. Ravenel from the sputum of a consumptive woman in September, 1899. As a further indication of its degree of virulence, it may be noted that two guinea-pigs were inoculated, subcutaneously, December 18, 1901, each with 1 c.c. of a standard suspension of this culture.

One guinea-pig died March 8th and the other March 20th, of generalized tuberculosis. Two rabbits were also inoculated December 16, 1901, each with 2 c.c. of the same suspension. Both died suddenly in June, one on the 3d and the other on the 10th, from having been given improper food. Both were free from all evidence of tuberculosis and showed no alteration excepting diffuse redness of the intestines.

These experiments tend strongly to show that cattle may be refractory to enormous quantities of tubercle bacilli from human sputum when injected into the blood beneath the skin, into the peritoneal cavity or into the lungs ; and the result upon one of the animals (the bull) indicates that after such treatment the resistance to virulent culture of bovine origin may be increased.

An experiment was inaugurated in March of this year, to again, and more definitely, test the immunizing value of repeated intravenous inoculations of cultures of sputum tubercle bacilli not virulent for cattle. For the purpose of this experiment four young cattle were used, as follows: A black and white bull, sixteen months old (46066); a red heifer, twelve months old (45068) ; a red heifer, fifteen months old (45067), and a red heifer, eleven months old (45071). All were tested with tuberculin and were proven to be free from tuberculosis. They were divided into two groups of two each as nearly equal as possible in respect to age, size, and general condition. The animals of one group were inoculated intravenously seven times between March 24th and June 2d, with gradually increasing quantities of from 10 c.c. to 25 c.c. of a standard suspension of a culture of sputum tubercle bacilli. In all, 125 c.c. of this suspension were administered, representing about 0.16 gramme of tubercle bacilli.

Each of the four animals in this experiment—the two that had been vaccinated (45066 and 45068) and the two kept as controls (45067 and 45071)—was inoculated July 29th by injecting into the trachea 10 c.c. of a standard suspension of bovine tubercle bacilli (culture H) known to be virulent for cattle. The intratracheal method of inoculation was used, because it furnished a means of conveying tubercle bacilli into the organs most frequently infected in nature and in a manner unattended by disturbance of function or with material traumatism. It seemed to give a mode of infection closely resembling the natural one. One of the vaccinated cattle (45068) was killed October 4th. A searching post-mortem examination revealed all of the organs, including their lymphatic glands and covering membranes, to be free from all evidence of disease, with the exception of a slight fibrous thickening of the wall of the jugular vein at the point of vaccination. At the site of the intratracheal inoculation of July 29th there was no mark, and the mucous membrane lining the trachea was entirely normal.

A control heifer (45071) killed October 8th showed the following upon post-mortem examination : At the point of inoculation, upon the outside of the trachea and beneath the skin, there was a globular abscess about three-quarters of an inch in diameter, containing cheesy pus. The mucous membrane of the trachea showed a number of small, reddish elevations (tubercular) below the point of inoculation. The lungs were studded upon the surface and upon cross section with grayish nodules one-quarter to one-half an inch in diameter, the centres of which were caseous. These tubercles were evenly distributed in both lungs and roughly averaged from one to one and one-half inches apart. They could be plainly seen and felt through the transparent pleura. The apex of the right lung contained a caseous area two inches in diameter, which was made up of many adjacent small tubercles. The bronchial and mediastinal lymphatic glands were enlarged and contained cheesy areas from one-sixteenth to one-third of an inch in diameter. The post-pharyngeal lymphatic glands were enlarged to the size of an egg, hyperæmic, and on section showed numerous caseous areas.

The second vaccinated animal (45066) was killed October 16th. At the two points of insertion of the needle when the animal was inoculated, July 29th, there were two somewhat hard, globular fibrous thickenings one-quarter to three-fifths of an inch in diameter, respectively. Within the trachea, and occupying positions corresponding to these, were two very small (pin-head) grayish elevations in the mucous membrane. Upon section it was found that the upper of these small thickenings was made up of fibrous tissue, the lower (the smaller one) contained a focus of caseous material surrounded by thick, fibrous walls. The whole appearance was that of a closed process. No other lesions were found in any part of the body. All of the organs, their lymphatic glands and covering membranes, appeared to be quite normal. There was no thickening of the wall of the jugular vein at the point of vaccination.

The second control (unvaccinated) heifer (45067) was killed October 16th. The post-mortem report is as follows : Beneath the skin in the middle of the neck, at the point of inoculation, there was an abscess about two inches in diameter that contained cheesy pus. All of the inferior cervical and suprasternal lymphatic glands were enlarged to several times their normal volume and contained numerous areas of caseation. Within the trachea, from the point of inoculation down to its bifurcation and up to the glottis, the mucous membrane lining the ventral half of the tube was thickly studded with oblong, red, and evidently young and progressive tubercular growths. These formations were from one-sixth to one-half an inch long, and about two-thirds as wide; they stood above the surrounding surface from one-twelfth to one-half an inch. The post-pharyngeal lymphatic glands were enlarged to the size of a hen's egg and loaded with caseous material. The lungs contained many grayish nodules one-eighth to one-quarter of an inch in diameter. The smaller were grayish throughout, while the larger had yellow, cheesy centres. These nodules were not set so thickly as in the other control heifer (45071). They averaged from four to five inches apart, and were very evenly distributed throughout both lungs. The mediastinal and bronchial lymphatic glands were enlarged to twice their normal size and contained much caseous material. Many (about eighteen) of the lymphatic glands of the mesentery were enlarged and caseous. No alteration could be found in the mucous membrane or the walls of the intestine. The infection of the mucous membrane of the trachea above the point of inoculation appears to have been due to the carriage upward by coughing of some of the tubercle bacilli at the time of inoculation. It is well known that cattle habitually swallow their expectorations, and this may account for the infection of the post-pharyngeal and mesenteric lymphatic glands.

From the experiments here recorded we believe that we are justified in concluding:

1. That after repeated intravenous injections of cultures of tubercle bacilli from human sputum the resistance of young cattle to virulent tubercle bacilli of bovine origin may be increased to such an extent that they are not injured by inoculation with quantities of such cultures that are capable of causing death or extensive infection of cattle not similarly protected.

2. That by intravenous injection much larger quantities of culture of human sputum tubercle bacilli than are necessary to confer a high degree of resistance, or immunity, upon the vaccinated animal may be administered without danger to that animal.

We now have in progress uncomplete experiments upon a number of young cattle, some of which have been underway since last March, for the purpose of testing the duration of this immunity and the extent to which it is effective in protecting cattle against infection from natural sources. We have also started an experiment which we hope will throw light upon the open question as to the minimum quantities of culture of non-virulent tubercle bacilli that it may be necessary to administer in order to confer a serviceable degree of immunity, and, further, whether it may be possible to simplify the process of vaccination by successive injections of a few cultures of progressive degrees of virulence.

In conclusion, we wish to express our thanks to Dr. M. P. Ravenel and to Dr. H. C. Campbell; to the former for the originals of most of the culture used, and to both for general assistance during the progress of the experiments. We also wish to thank the authorities of the Veterinary Hospital and of the Pepper Clinical Laboratory of the University of Pennsylvania, who have generously furnished the State Live-stock Sanitary Board with a laboratory and with other facilities, without which its research work would have been impossible.

## Figures and Tables

**Figure f1:**
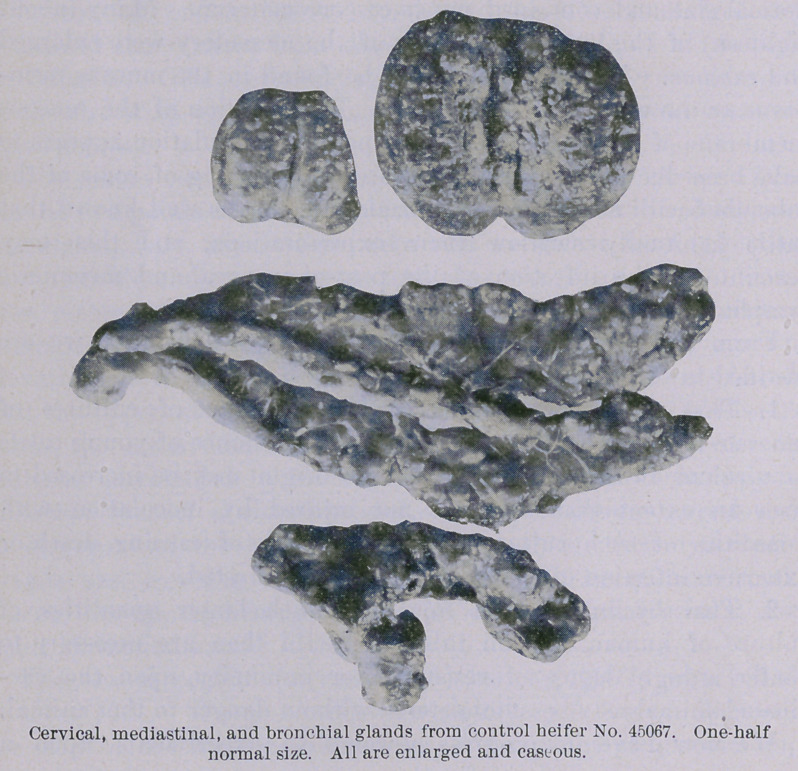


**Figure f2:**
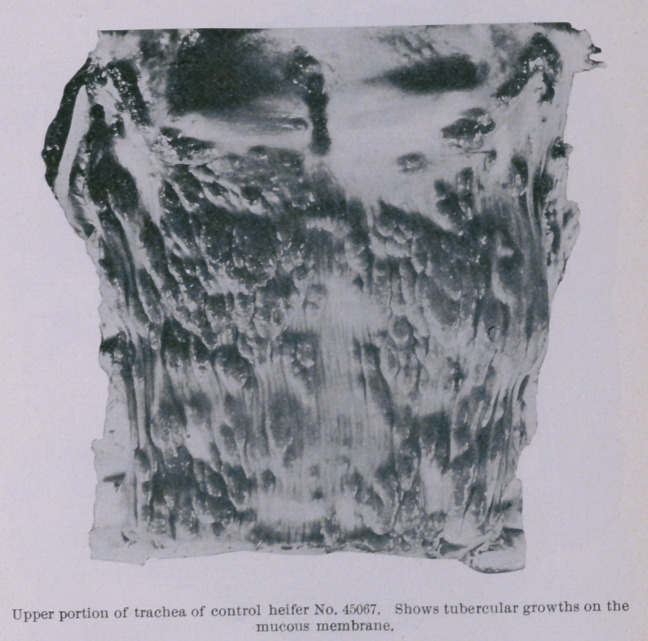


**Figure f3:**
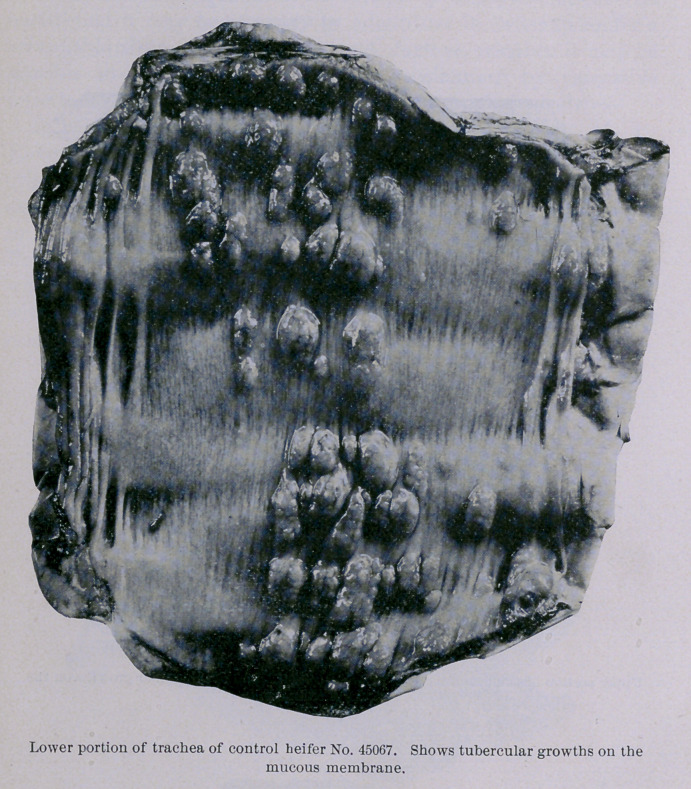


**Figure f4:**
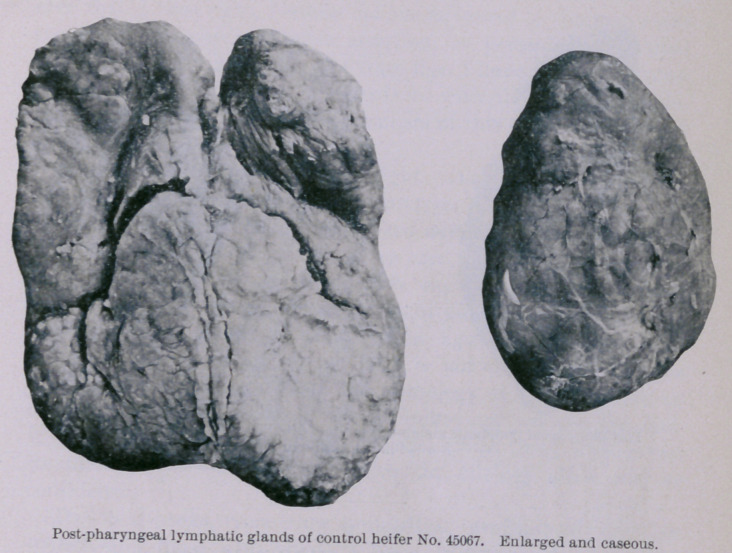


**Figure f5:**
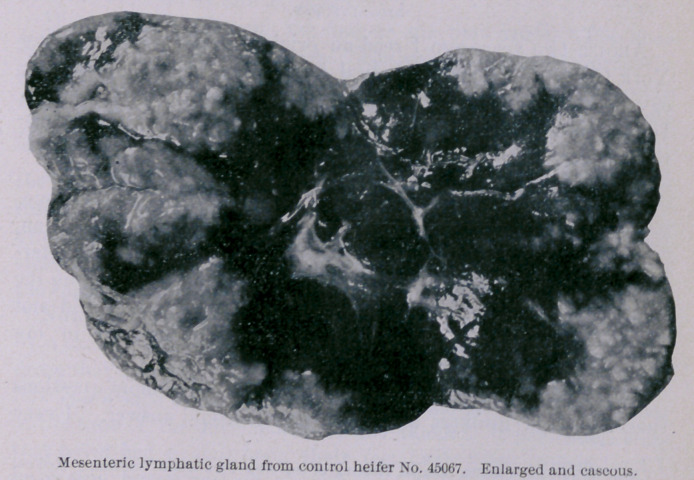


**Figure f6:**